# Exploring the Antioxidant Features of Polyphenols by Spectroscopic and Electrochemical Methods

**DOI:** 10.3390/antiox8110523

**Published:** 2019-10-31

**Authors:** Berta Alcalde, Mercè Granados, Javier Saurina

**Affiliations:** Department of Chemical Engineering and Analytical Chemistry, University of Barcelona, Martí i Franquès 1-11, 08028 Barcelona, Spain; berta.alcalde.96@gmail.com (B.A.); mgranados@ub.edu (M.G.)

**Keywords:** antioxidant index, differential pulse voltammetry, polyphenols, index correlation, structure-activity

## Abstract

This paper evaluates the antioxidant ability of polyphenols as a function of their chemical structures. Several common food indexes including Folin-Ciocalteau (FC), ferric reducing antioxidant power (FRAP) and trolox equivalent antioxidant capacity (TEAC) assays were applied to selected polyphenols that differ in the number and position of hydroxyl groups. Voltammetric assays with screen-printed carbon electrodes were also recorded in the range of −0.2 to 0.9 V (vs. Ag/AgCl reference electrode) to investigate the oxidation behavior of these substances. Poor correlations among assays were obtained, meaning that the behavior of each compound varies in response to the different methods. However, we undertook a comprehensive study based on principal component analysis that evidenced clear patterns relating the structures of several compounds and their antioxidant activities.

## 1. Introduction

Epidemiological studies have shown that antioxidant molecules such as polyphenols may help in the prevention of cardiovascular and neurological diseases, cancer and aging-related disorders [1,2,3,4]. Antioxidants act against excessively high levels of free radicals, the harmful products of aerobic metabolism that can produce oxidative damage in the organism [5]. Another protective effect attributed to polyphenols is their metal chelation ability, which decreases the potential toxicity of heavy metals [6].

Polyphenols are a large family of secondary metabolites of the plant kingdom, which comprises more than 8000 different compounds, and are especially abundant in fruits, vegetables and cereals. Polyphenols are often classified according to their molecular complexity into four main families that differ in the number of phenol rings and other structural elements, namely, [2,7] (i) phenolic acids, the simplest compounds, comprising the subclasses of hydroxybenzoic and hydroxycinnamic acids, which account for ca. 30% of total dietary polyphenols; (ii) flavonoids, the most abundant group representing 60% of human intake, consist of two aromatic rings linked by an oxygen heterocycle; and several flavonoid subfamilies are defined depending on the degree of oxidation and substitutions in the heterocycle; (iii) stilbenes, with a double-bond connecting two aromatic rings, have high nutritional significance despite being present in low quantities in the human diet; and (iv) lignans, a minor class of polyphenols consisting of two phenylpropane units.

Polyphenols in food products are highly relevant because they are associated with their sensorial and functional properties. It is well-known that some polyphenol families have a strong influence on the color, flavor, bitterness and astringency of foodstuffs [8]. With regard to the health aspects, polyphenols provide supplementary benefits such as anti-bacterial, anti-inflammatory, anti-allergic, antineoplastic and anti-thrombotic activities [3,4,5].

The research on natural antioxidants has increased significantly in recent years and dozens of new scientific articles have been published as it can be seen in various interesting reviews on this topic [9,10,11]. Although the reducing power and the anti-radical activity of food products are not unique to polyphenols, these compounds are the main sources of antioxidants in the human diet. Several classical antioxidant assays are currently used to estimate the total phenolic content and antioxidant capacity of fruits and vegetables. These assays are based on chemical reactions involving hydrogen atom transfer (HAT) or single electron transfer (SET). Oxygen radical absorbance capacity (ORAC) is an example of a HAT assay, Folin-Ciocalteau (FC) and ferric reducing antioxidant power (FRAP) are SET assays, whereas trolox equivalent antioxidant capacity (TEAC) assays involve both SET and HAT mechanisms [12,13,14]. Results achieved from each of these indexes are seldom comparable, especially when comparing SET and HAT assays. The lack of solid equivalences between the indexes may be due to (i) the mechanisms involved; (ii) the different reference standards used for expressing the antioxidant activity (e.g., gallic acid, trolox, quercetin, etc.); (iii) the fact that each particular compound has different sensitivities towards each index; and (iv) the great complexity of food matrices, which are often a source of interferences and matrix effects [14].

Apart from multiple chemical issues, researchers have expressed serious concerns about the suitability of food indexes to describe the actual in vivo antioxidant activity. Other concerns include that in vitro reaction conditions (e.g., the concentration of free radicals, pH, occurrence of multiple endogenous compounds, etc.) can hardly be extrapolated to humans [15]. Additionally, the bioavailability and metabolism of dietary polyphenols should also be taken into account as the concentrations (and chemical forms) found in biological fluids differ significantly from those present in food products [16,17,18]. Nonetheless, chemical assays are still applicable for preliminary screenings of the antioxidant ability of food products because of their simplicity and low cost.

Further insights into the antioxidant properties of molecules can also be gained by electrochemical techniques [19,20,21,22], especially by using cyclic, square-wave and differential pulse voltammetry (DPV). Anodic signals contain relevant information about how easily compounds can be oxidized as well as the oxidation mechanisms. Quantitative data about the overall polyphenolic content can be estimated from the current intensities of the voltammetric scans [23,24] while individual concentrations of targeted polyphenols can be determined in combination with separation techniques such as liquid chromatography [24,25,26]. The comparison of voltammetric measurements with spectroscopic indexes has been investigated by several authors, for example, in the assessment of the antioxidant capacity of oenological tannins [27], berry fruits [28], propolis extracts [29], red wines [30], green tea [31] and hops [32]. In addition, electronic voltammetric tongues consisting of an array of modified graphite-epoxy composites plus a gold microelectrode have been used to predict the FC index of wines [33]. Based on these studies, electrochemical techniques are viewed as very simple and promising approaches for predicting the antioxidant activity of food related products.

This work aims to compare the reducing, anti-radical and voltammetric behavior of various polyphenols to understand the relationship between their chemical structures and their antioxidant activities. FC was used to determine the total polyphenolic content, 2,4,6-tripyridyl-S-triazine (TPTZ) was used to estimate FRAP, and 2,2′-azino-bis(3-ethylbenzothiazoline-6-sulfonic) acid (ABTS) and 2,-diphenyl-1-picrylhydrazyl (DPPH) was used to quantify TEAC indexes. In addition, DPV with screen-printed carbon electrodes was applied to carry out an electroanalytical characterization. Compounds were selected to encompass the diversity of the main polyphenolic families, including phenolic acids, flavonoids and stilbenes. Special attention was paid in the case of hydroxybenzoic acids to assess the influence of the number and position of hydroxyl groups on the antioxidant activity. Correlation studies and chemometric methods, such as principal component analysis were applied to better understand the overall behavior of analytes and indexes.

## 2. Materials and Methods 

### 2.1. Reagents and Solutions

Representative polyphenols selected for this study were as follows: 3-hydroxybenzoic acid (99%), 4-hydroxybenzoic acid (≥99%), 2,4-dihydroxybenzoic acid (97%), 3,5-dihydroxybenzoic acid (97%), 2,3-dihydroxybenzoic acid (99%), 2,3,4-trihydroxybenzoic acid (97%), 2,5-dihydroxybenzoic acid (98%), 2,4,-dihydroxybenzoic acid (97%), 2,4,6-trihydroxybenzoic acid (90%), 3,4-dihydroxybenzoic acid (≥99), and gallic acid (97.5%), which were purchased from Sigma Aldrich (St Louis, MO, USA); luteolin and 6-hydroxy-2,5,7,8-tetramethylchroman-2-carboxylic acid (trolox) were purchased from Carbosynth (Berkshire, UK); quercetin (>99%) from Merck (Darmstadt, Germany); and resveratrol (>99%) and catechin (>95%) were provided by TCI (Zwijndrecht, Belgium). The solvents used for the preparation of stock and working solutions were dimethylsulfoxide (DMSO), (Merck, Darmstadt, Germany), methanol (UHPLC-Supergradient, Panreac, Barcelona, Spain), ethanol (HPLC grade, Merck, Darmstadt, Germany) and Milli-Q water purified using an Elix 3 coupled to a Mili-Q system. Stock solutions of each compound were prepared at 1000 mg L^−1^ in DMSO. Working solutions for calibration were prepared in the range of 0.2–5 mg L^−1^ by proper dilution with ethanol/water (1:1, v:v).

The reagents used for the spectrophotometric indexes were as follows: formic acid and potassium peroxodisulfate from Sigma Aldrich; hydrochloric acid (32%, w:w), sodium hydroxide, Fe(III) chloride, sodium carbonate and disodium hydrogen phosphate from Merck; FC reagent from Panreac; 2,2′-azino-bis(3-ethylbenzothiazoline-6-sulfonic) acid (ABTS), 2,-diphenyl-1-picrylhydrazyl (DPPH), and 2,4,6-tripyridyl-S-triazine (TPTZ) from Alfa Aesar (Kandel, Germany). Reagents for the preparation of the background buffer solution for voltammetry were acetic acid (>99%, Sigma-Aldrich) and sodium acetate (analytical grade, Merck, Darmstadt, Germany).

### 2.2. Instruments

A double beam Perkin Elmer UV/Vis/NIR Lambda 19 spectrophotometer (Waltham, MA, USA) was used to measure antioxidant and anti-radical indexes. Standard and reagent blank solutions were located in the sample and reference holders, respectively. QS quartz high performance cuvettes (10 mm optical path) from Hellma Analytics (Jena, Germany) were used.

Electrochemical studies were performed with a μAutolab system Type (III) (EcoChemie, Utrecht, The Netherlands) attached to a 663VA stand (Metrohm, Herisau, Switzerland). In all cases, a conventional cell was used with Ag|AgCl|KCl (3 mol L^−1^) as the reference electrode, platinum wire as the auxiliary electrode and a screen-printed carbon DRP-110 (Dropsens, Oviedo, Spain) as the working electrode in DPV mode. Data was acquired with a personal computer using the GPES 4.9 software (EcoChemie, Utrecht, The Netherlands).

### 2.3. Analytical Methods

#### 2.3.1. FC Assay

One mL of water and 250 μL of FC reagent were placed in an amber glass vial. After 8 min, 75 μL of 7.5% (w:v) sodium carbonate aqueous solution and appropriate volumes of polyphenols were added to the vial to get concentrations in the range of 0.2–5 mg L^−1^. Water was then added to obtain a final volume of 5 mL. The reaction was developed for 2 h and the absorbance was recorded at 765 nm using the reagent blank as the reference.

#### 2.3.2. FRAP Assay

FRAP reagent was prepared by mixing 20 mmol L^−1^ FeCl_3_, 10 mmol L^−1^ TPTZ (containing 50 mmol L^−1^ HCl) and 50 mmol L^−1^ formic acid solution in the proportion of 1:2:10 (v:v:v). The reaction was developed with 300 μL of FRAP reagent and appropriate volumes of each polyphenol standards (to provide concentrations in the range of 0.2–5 mg L^−1^), and were diluted with Milli-Q water to obtain a final volume of 2.5 mL. The absorbance resulting after 5 min of reaction was measured at 595 nm using the reagent blank as the reference.

#### 2.3.3. ABTS Assay

A stock solution of the cation radical species referred to as ABTS•^+^ was generated with 20 mL of 7 mM ABTS and 350 μL of 140 mM potassium peroxodisulfate. The mixture was kept in the dark for at least 16 h before use and was stable for 1 week when stored at 4 °C. A working solution was prepared daily by diluting 300 μL of ABTS•^+^ stock solution in 12 mL of ethanol. The assay was carried out by mixing 1.5 mL of ABTS•^+^, the required volume of polyphenol standard and ethanol up to 2.5 mL. Absorbance values were measured at 734 nm after 25 min of reaction time, using the ABTS•^+^ blank as the reference.

#### 2.3.4. DPPH Assay

A 0.2 mM DPPH stock solution in 50 mL ethanol was prepared and was kept in the dark for 2 h. The assay was carried out in an amber glass vial by mixing 2 mL of the DPPH solution, 1.6 mL 0.1 M phosphate buffer (pH 7.4), the required volume of the standard (providing concentrations in the range 0.2 to 5 mg L^−1^), and ethanol up to 4 mL. The solution was mixed and kept in the dark for 45 min. The absorbance was then measured at 517 nm using the reagent blank as the reference.

#### 2.3.5. Voltammetric Assay

The required volume of standards was added to 25 mL of supporting electrolyte (0.1 M sodium acetate-acetic acid buffer at pH 5) in the electrochemical cell. Differential pulse voltammograms were recorded in the range from −0.2 to +0.9 V. Other conditions included a scan rate 0.1 mV s^−1^, modulation time of 0.05 s, and interval time of 0.5 s.

### 2.4. Data Analysis

Excel (Microsoft, Redmond, WA, USA) was used for preliminary correlation and statistical studies. Principal component analysis (PCA) using the PLS-Toolbox (working under MATLAB, Applied Chemometrics, Inc, PO Box 100, Sharon, MA, USA) was applied to a global characterization of selected polyphenols according to spectrophotometric indexes and voltammetric data. The data matrix consisted of 15 rows (selected polyphenols) and 5 columns (slopes from FC, FRAP, ABTS, DPPH, and DPV methods). Data was autoscaled to equalize the influence of each variable in the model.

## 3. Results and Discussion

Fifteen polyphenols were chosen as the model compounds to be compared in order to investigate the relationships between the molecule structure and the antioxidant activity. Various food indexes were assessed with regard to their redox, anti-radical and electrochemical properties. Calibration curves from FC, FRAP, ABTS, DPPH and DPV methods were obtained for each polyphenol as described in Section 2.3. In all cases, the working range was 0.2–5 mg L^−1^ and the resulting sensitivities, in terms of mAU mol^−1^, expressed the antioxidant ability of each molecule. In the case of TEAC indexes based on ABTS and DPPH reagents, it should be noted that the slopes were negative as the addition of polyphenols decreased the amount of free radical reagent, thus resulting in a decrease in absorbance with increasing concentration.

The results summarized in Table A1 (Appendix A) show that several compounds such as 3-hydroxybenzoic, 4-hydroxybenzoic, 2,4-dihydroxybenzoic and 2,4,6-trihydroxybenzoic acids displayed, in general, poor activity in all the assays. Other compounds, such as gallic acid, quercetin or luteolin, showed high responses for most of the indexes. Additionally, it was observed that various isomers with differing hydroxylation positions presented quite dissimilar behavior. For instance, 3,5-dihydroxybenzoic acid demonstrated low sensitivities while 2,3-dihydroxybenzoic acid exhibited higher values. Similar results were found when comparing 2,4,6-trihydroxybenzoic acid and gallic acid. It was thus concluded that, apart from the degree of hydroxylation, the antioxidant power depended on structural issues.

**Figure 1:**
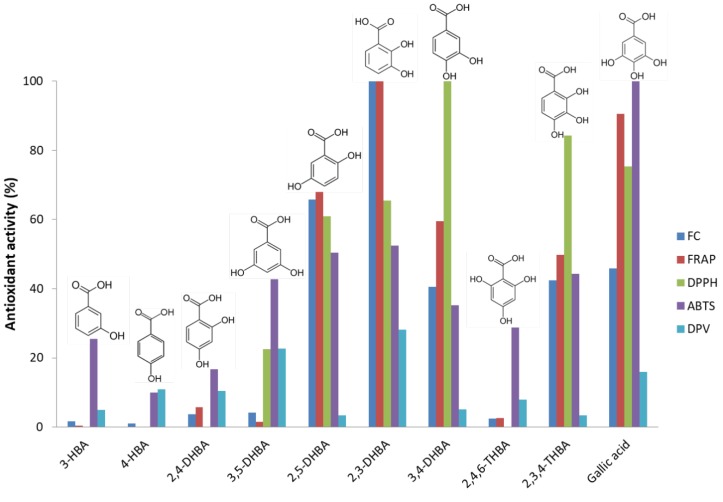
Comparison of food indexes and electrochemical assay for the series of hydroxybenzoic acids. Data corresponds to the normalized slopes of the calibration curves of each analyte and each method. Compound assignation: 3-HBA, 3-hydroxybenzoic acid; 4-HBA, 4-hydroxybenzoic acid; 2,4-DHBA, 2,4-dihydroxybenzoic acid; 3,5-DHBA, 3,5-dihydroxybenzoic acid; 2,5-DHBA, 2,5-dihydroxybenzoic acid; 2,3-DHBA, 2,3-dihydroxybenzoic acid; 3,4-DHBA, 3,4-dihydroxybenzoic acid; 2,4,6-THBA, 2,4,6-trihydroxybenzoic acid; 2,3,4-THBA, 2,3,4-trihydroxybenzoic acid; Gallic acid, 3,4,5-trihydroxybenzoic acid (chemical structures are also given). Method assignation: FC, Folin-Ciocalteau; FRAP, Ferric Reducing Antioxidant Power; DPPH, 2,-diphenyl-1-picrylhydrazyl; ABTS, 2,2′-azino-bis(3-ethylbenzothiazoline-6-sulfonic) acid; DPV, differential pulse voltammetry.

In order to gain more information on the role of the hydroxylation on the antioxidant properties of molecules we focused our study on the benzoic acid series because of the simplicity of their structure. The higher activities seemed to be associated with the presence of more than one hydroxyl group conveniently oriented in ortho or para positions while those in meta or monohydroxylated species were less active. The strongest reducing agents according to FC assay were dihydroxy- and trihydroxybenzoic acids with *o*- and *p*-configurations, while those not following this pattern were less efficient. Similar behaviors were observed for FRAP and DPPH assays. The results of ABTS method were more erratic and independent of the stereochemistry of the molecules. A comparison of the normalized antioxidant activity of all assays (Figure 1) also showed some subtle differences in the overall behavior of the isomers. The structure of each molecule has been included for a better interpretation of results.

**Figure 2:**
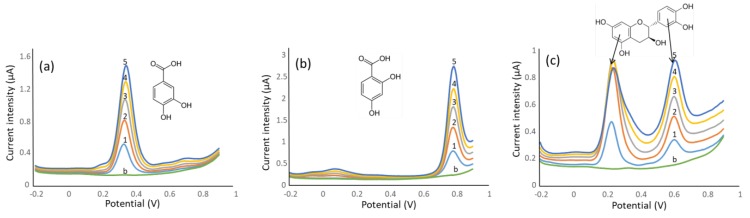
Differential pulse voltammograms recorded in the range −0.2 to +0.9 V vs. Ag|AgCl|KCl reference electrode for three representative examples: (**a**) 3,4-dihydroxybenzoic acid; (**b**) 2,4-dihydroxybenzoic acid; (**c**) catechin. Assignation: b = blank; 1 to 5, standard additions 1 × 10^−5^ g L^−1^ each.

The behavior of the molecules in the process of oxidation, which is closely related to their antioxidant power, can be better interpreted from electroanalytical studies by DPV. The representative examples depicted in Figure 2 indicated that molecules with more than one hydroxyl groups in *o*- and *p*- configurations displayed anodic processes at quite low potentials, with peak maxima at ca. 0.2–0.3 V (vs. Ag|AgCl|KCl reference electrode), thus indicating that these substances are highly prone to undergoing oxidation (see the example of 3,4-dihydroxybenzoic acid in Figure 2a). Conversely, meta or monohydroxylated species required higher potentials to be oxidated, with anodic bands at 0.65–0.85 V, which correspond to less labile species in oxidation reactions (see voltammograms of 2,4-dihydroxybenzoic acid in Figure 2b). A DPV scan of catechin is depicted for illustrative purposes (see Figure 2c). This molecule, with 2 independent dihydroxyphenyl moieties with *o*- and *m*- configurations, showed two oxidation peaks at 0.22 and 0.72 V attributed to the respective patterns mentioned above.

Apart from the oxidation potential, the current intensity as a function of the concentration was another interesting feature closely related to the antioxidant power. It was considered that species with higher sensitivities could be stronger antioxidants. Thus, quercetin, luteolin and resveratrol acted as powerful substances while some hydroxybenzoic acids (e.g., 3-hydroxybenzoic, 3,4-dihydroxybenzoic and 2,5-dihydroxybenzoic acids) displayed poor activities.

To summarize, anodic potentials provide qualitative information about the lability of the substances in (electro)chemical oxidant conditions, thus indicating the threshold from which they will act as antioxidants (i.e., the highest labilities have the lowest potential). In this regard, we guessed that in a mixture of polyphenols, compounds with the lowest potential will be the first to start acting as antioxidants while those with higher potential will remain in reserve until the others are consumed. In contrast, the slope of the calibration curves provides information on the quantitative power of the molecules to combat the presence of oxidant species in the medium.

**Figure 3:**
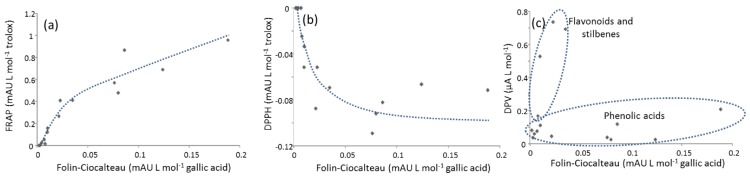
Correlation studies among Folin-Ciocalteau (FC) and other methods. Scatter plots represent the slopes of the calibration curves of each compound in the following cases: (**a**) FC vs. Ferric Reducing Antioxidant Power (FRAP); (**b**) FC vs. 2,-diphenyl-1-picrylhydrazyl (DPPH); (**c**) FC vs. Differential Pulse Voltammetry (DPV). Method assignation: see Figure 1.

A preliminary comparison of data from the different indexes was based on correlation studies. For FC vs. FRAP, the data was certainly correlated (*r* = 0.92), thus suggesting that the reduction of respective Mo(VI) and Fe(III) complexes occurs in analogous circumstances (see Figure 3a). Hence, the two indexes provided similar information regarding the reducing ability of each polyphenol. In the other cases, poorer correlations were obtained, meaning that the data was more dissimilar. However, a more thorough inspection of the scatter plots of various indexes revealed additional details. For instance, FC vs. DPPH (Figure 3b) showed that compounds with high reducing power also display high radical scavenging activity and vice versa. Figure 3b, however, suggested saturation in the anti-radical activity of the most powerful compounds, while molecules with low or moderate activities displayed a more linear relationship. The scatter plot of FC vs. DPV (Figure 3c) showed two characteristic trends depending on the nature of compounds. For instance, flavonoids and stilbenes were more electroactive than phenolic acids. In the case of FC vs. ABTS, the results were more dispersed, although, in general, the most (and less) active compounds were the same in the two systems. Analogous examples considering FRAP instead of FC (results not shown) were studied and the conclusions that were drawn are similar to those presented here.

### 3.1. Comparison of Indexes by PCA

**Figure 4:**
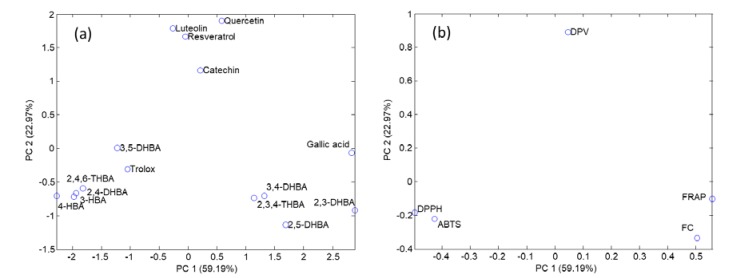
Principal component analysis for the evaluation of the antioxidant activity of model compounds as a function of spectroscopic and electrochemical assays: (**a**) Plot of scores; (**b**) Plot of loadings. Compound and method assignation: see Figure 1.

A more comprehensive evaluation of the data from spectroscopic and voltammetric experiments was carried out by PCA. As indicated in the experimental section, an autoscaled model was built to minimize the impact of the differences in magnitude and amplitude of indexes on the description of samples and variables. The first and second principal components (PC1 and PC2) explained 59.2 and 23.0% of the data variance, respectively. The scatter plot of scores of PC1 and PC2 showed three characteristic patterns regarding the distribution of the samples (see Figure 4a). The group of compounds located to the right side corresponded to di- and trihydroxybenzoic acids having *o*- and *p*- oriented hydroxyl groups. Compounds on the left corresponded to benzoic acids with 1 hydroxyl group or several in *m*- positions. The group in the middle consisted of flavonoids and resveratrol and exhibited intermediate features as they contained both *o*- and *m*- moieties in their structures.

The plot of loadings provided information about the correlation among variables (Figure 4b). It was found that PC1 mainly described the overall antioxidant activity, with spectroscopic methods with a positive slope (FC and FRAP) to the right and those with a negative slope (DPPH and ABTS) to the left. PC2 was used for modeling differences among spectroscopic (bottom) and voltammetric methods (top), and indicated that correlations among colorimetric indexes and DPV were, in general, poor.

The simultaneous interpretation of scores and loadings suggested that, from PC1, compounds located to the right were stronger antioxidants from an overall point of view (basically considering their reducing, anti-radical abilities). PC2 discriminated chemical and electrochemical information. It was observed that the most sensitive electroanalytical species were located at the top while those with smaller slopes were at the bottom. As a result, it was concluded that PC1 mainly explained the polyphenolic behavior according to the spectroscopic indexes and PC2 modeled the voltammetric features of the compounds.

## 4. Conclusions

The estimation of the antioxidant ability of food products and identifying the ability of bioactive compounds to reduce oxidative stress are increasingly studied due to their health implications. Unfortunately, information about the antioxidant activity of natural compounds such as polyphenols is sometimes confusing and data from food indexes regarding their reducing or anti-radical power are seldom coherent. Apart from using different reference compounds for expressing such activity (e.g., gallic acid equivalents in FC, trolox equivalents in FRAP or TEAC, or quercetin or rutin in flavonoid indexes), the sensitivities of compounds in the assays are different. This paper has untangled some of the discrepancies that are often encountered in the study of the antioxidant activity of molecules by using a global approach that combines data from various food indexes and electrochemical studies. The PCA model revealed three distinct patterns depending on the number and orientation of hydroxyl groups in the molecules. *p*- and *o*-dihydroxyphenyl molecules were clearly discriminated from their *m*- counterparts. Besides, electrochemical data explained both qualitative and quantitative aspects of the antioxidant power of molecules.

In short, we assume that the oxidation of molecules by chemical, radical and electrochemical mechanisms is easier in the case of dihydroxyphenyl moieties with *o*- and *p*- orientations. Hence, the corresponding benzoquinones can be easily formed as a first oxidized species. In the case of phenyl moieties with single hydroxylation, the oxidation mechanism also leads to a benzoquinone via a more complex mechanism. The reaction is not as favored as in the previous case and stronger oxidants or higher potential may be required. Finally, processes involving dihydroxyphenols *m*-substituted have not been so well described but the reactions seem to be more complex and their antioxidant activity may be more limited.

## Appendix A

**Table A1:** Comparison of spectroscopic antioxidant indexes and electrochemical data for selected model polyphenols. Values given in the table correspond to slopes of the corresponding calibration curves of each analyte and each method under optimal conditions.

Polyphenol	Folin-Ciocalteau (AU L mol^−1^ at λ = 765 nm)	FRAP (AU L mol^−1^ at λ = 595 nm)	DPPH (AU L mol^−1^ at λ = 517 nm)	TEAC (AU L mol^−1^ at λ = 734 nm)	Voltammetry (nA L mol^−1^)
3-hydroxybenzoic acid	0.4	0.5	0	−9.7	35.9
4-hydroxybenzoic acid	0.3	0.2	0	−3.8	80.3
2,3-dihydroxybenzoic acid	29.0	149	−11.0	−22.2	207
2,4-dihydroxybenzoic acid	1.2	8.3	0	−8.7	76.3
2,5-dihydroxybenzoic acid	19.1	106	−10.2	−21.5	24.9
3,4-dihydroxybenzoic acid	11.8	87.8	−16.8	−15.6	37.5
3,5-dihydroxybenzoic acid	1.2	2.3	−3.8	−18.5	167
2,3,4-trihydroxybenzoic acid	13.6	81.1	−15.6	−21.1	24.4
2,4,6-trihydroxybenzoic acid	0.8	4.8	0	−17.4	58.5
gallic acid	6.2	150	−14.0	−48.7	118
trolox	2.6	39.6	−8.4	−22.0	110
catechin	6.3	73.5	−24.6	−35.0	45.2
quercetin	11.9	139	−23.5	−47.7	692
luteolin	6.5	117	−14.7	−21.3	735
resveratrol	2.3	27.6	−11.7	−44.6	59.0
